# Impact of Polymers on Sand Sedimentation Characteristics of Shale Oil-Produced Fluid

**DOI:** 10.3390/ma18102269

**Published:** 2025-05-14

**Authors:** Yongbin Shang, Qiaosheng Zhang, Wanrui Li, Tian Gao, Ruhao Zhao, Lan Bai, Xiaoming Luo

**Affiliations:** 1 Changqing Engineering Design Co., Ltd., China Petroleum and Natural Gas Corporation, Xi’an 710018, China; 2 National Engineering Laboratory for Exploration and Development of Low-Permeability Oil & Gas Fields, China Petroleum and Natural Gas Corporation, Xi’an 710018, China; 3 College of Pipeline and Civil Engineering, China University of Petroleum (East China), Qingdao 266580, China

**Keywords:** shale oil, polymer, sand sedimentation characteristics, flocculation–sedimentation, supercritical water oxidation

## Abstract

The introduction of polymers has significantly altered the properties of sand particles in shale oil production fluids, leading to a more complex sedimentation mechanism. However, the specific ways in which polymers influence sand sedimentation dynamics remain poorly understood. In this study, Soxhlet extraction and supercritical water oxidation techniques were employed to compare the particle size distribution of polymer-containing sand with that of actual sand. The results show that sand sedimentation in polymer-containing shale oil production fluids involves two mechanisms: gravity-dominated single-particle sedimentation and polymer-induced multi-particle flocculation–sedimentation. Additionally, polymers induce both flocculation–sedimentation and hindering effects. Specifically, the water content and temperature can promote single-particle sedimentation and flocculation–sedimentation of the sand particle group by adjusting the rheology, polymer content, and stability of the production fluid. In this experimental study, the sedimentation rates of the two processes were increased by 38.05% and 54.76%, respectively. Based on these findings, the sedimentation characteristics of sand particles in production fluids under the influence of polymers were obtained, offering valuable insights for the management and control of sand in polymer-containing shale oil production fluids.

## 1. Introduction

Hydraulic fracturing technology improves the feasibility of the development of shale oil reservoirs, while polymer flooding improves the flow ratio by increasing the viscosity of the displaced phase, thereby significantly enhancing recovery efficiency [1,2,3]. However, HPAM-containing displacement fluids cause shear erosion effects on formation fractures, leading to the production of a large amount of fine sand particles, along with the produced fluid [4,5], forming a complex oil–water–sand–polymer system, with multiphase synergistic effects. This easily leads to issues, such as pipeline blockages [6,7,8] and increased internal corrosion [9,10,11]. Therefore, understanding the sedimentation mechanism of sand particles in polymer-containing production fluids and maintaining their suspension stability is key to ensuring the safe and efficient operation of multiphase gathering systems [12].

Since Stokes established the classical sedimentation model in 1851 [13], researchers have conducted numerous experiments and simulations on particle sedimentation characteristics [14,15]. In recent years, sand particle sedimentation models have been continuously refined. Researchers have developed sedimentation prediction models considering particle morphology characteristics, using computational fluid dynamics (CFD) with the discrete element method (DEM) [16,17]. These models indicate that irregular shapes significantly affect the sedimentation trajectory, coordination structure, and wake evolution behavior of sand particles. Zhu et al. [18] investigated the heat transfer and sedimentation of fine particles in porous media using CFD–DEM simulations. In experimental research, due to the opacity of crude oil, detailed tracking of the sand sedimentation process is limited. Researchers have mainly focused on the factors influencing sand sedimentation characteristics. Yan et al. [19] compared the sand particle size distribution in oil before and after sedimentation, confirming that increasing the temperature and reducing the heavy oil viscosity facilitate sand particle sedimentation. Although the static sedimentation experiments by Alajmi et al. [20] and Zhang et al. [21] verified the key role of the temperature–viscosity relationship, they overlooked the impact of fluctuations in the sand concentration and water content on true gathering systems [22]. In engineering practice, oil fields commonly use methods such as cyclones, gravity settling ponds, and high-precision filtration equipment for sand removal [23,24]. However, when dealing with high polymer and high viscosity production fluids, the effectiveness of such treatments is limited [25]. The reason is that the impact of polymer–particle interactions was not considered in the abovementioned sand particle sedimentation studies, leading to significant prediction deviations and an inability to accurately characterize the sand sedimentation characteristics of polymer-containing shale oil production fluids.

In the field of polymer–particle interaction research, researchers have primarily focused on the hindering and adsorption effects of polymers on particles. Numerous theoretical models provide the theoretical foundation for describing the rheological characteristics of polymer-containing fluids [26,27], effectively characterizing the viscoelastic properties of HPAM and other polymer solutions [28]. Experimental studies have shown that HPAM significantly alters the flow characteristics of the continuous phase, thereby exerting a significant hindering effect on the motion of dispersed-phase particles, such as oil droplets [29,30]. On the other hand, HPAM can also adhere to particle surfaces through hydrogen bonding and as a result of van der Waals forces [31], thus affecting the interactions between the particles. When HPAM and other polymers are inside dispersed water droplets, they can be co-adsorbed with other small particles and asphaltenes at the droplet interface [32,33,34,35], which can, to some extent, facilitate the synergistic sedimentation of water droplets and small particles. When HPAM and other polymer solutions are involved in the continuous phase, the adsorption effect of the polymer can also induce aggregation and adhesion between dispersed particles, thereby changing the equivalent density of the dispersed phase particles and achieving flocculation–sedimentation [36,37,38]. Furthermore, researchers have explored and assessed the characteristics of polymer–particle materials using machine learning [39] and magnetic induction eddy current methods [40]. However, the abovementioned studies primarily focus on the qualitative description of microscopic mechanisms and lack quantitative analysis of the sedimentation characteristics of polymer-containing particles from a macroscopic perspective. Moreover, they fail to reflect the true particle size distribution characteristics after removing the polymer, and cannot further clarify the sand particle sedimentation mechanism under the influence of polymers.

To address this issue, supercritical water oxidation (SCWO) technology demonstrates unique treatment advantages [41,42]. When water is above the critical point, its dielectric constant drops sharply, and the concentration of free radicals increases, enabling the efficient degradation of organic components [43,44]. Studies show that SCWO technology can effectively degrade organic pollutants, such as polymers in polymer-containing oil sludge (PCOS), transforming them into gas, oil, liquid, and solid products [45]. Peng et al. [46] found that supercritical water exhibits excellent solubility for PCOS and can achieve residue-free treatment of PCOS under supercritical conditions (400–700 °C, 23 MPa). Chen et al. [47] confirmed that under SCWO conditions at 500 °C, the total organic carbon (TOC) removal efficiency from oil-based drill cuttings can reach 89.2% within 10 min. This characteristic leads to a new approach for the treatment of the “polymer–sand” composite: SCWO treatment disrupts the polymer adhesion layer, restoring the intrinsic sedimentation characteristics of sand particles.

Based on the abovementioned analysis, it is clear that existing sand particle sedimentation studies mainly focus on net particle sedimentation models that ignore the influence of polymers, while polymer–particle interaction studies also neglect the true particle size distribution of sand particles after eliminating the influence of polymers, failing to further clarify the impact mechanism of polymers on sand particle sedimentation. Therefore, this paper constructs a temperature-controlled sand sedimentation experimental system and combines Soxhlet extraction with supercritical water oxidation technology to conduct a comparative study of the polymer-containing sand particle size distribution and the true sand particle size distribution. It analyzes the impact of polymers on the sand particle size distribution, providing theoretical support for sand particle management in regard to polymer-containing shale oil production fluids.

## 2. Experiments

### 2.1. Experimental Setup

(1) Temperature-controlled sand sedimentation experimental system

**Figure 1:**
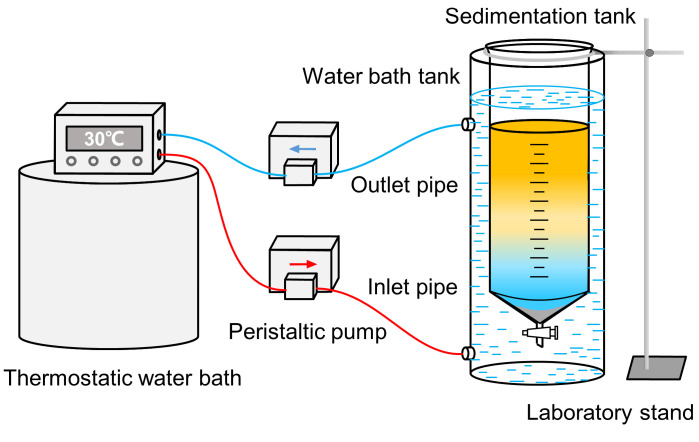
Temperature-controlled sand sedimentation experimental system.

The temperature-controlled sand sedimentation experimental system consists of a sedimentation tank, a water bath tank, a thermostatic water bath, a peristaltic pump, an inlet pipe, an outlet pipe, and a support frame, as shown in Figure 1. The sedimentation tank is a high borosilicate glass separating funnel, with a diameter of 12 cm and a height of 60 cm, supported by a frame and suspended at the center of the water bath tank. Two Kamoer DIP1500 peristaltic pumps (Kamoer Fluid Tech Co., Ltd., Shanghai, China) are connected to the water bath tank and the thermostatic water bath through inlet and outlet pipes, forming a thermostatic water bath circulation system. The SHP 501 thermostatic water bath (Sunny Hengping Scientific Instrument Co., Ltd., Shanghai, China) has a temperature control accuracy of ±0.5 °C, ensuring that there are no temperature differences in the various regions within the sedimentation tank.

(2) Sand sedimentation treatment system

**Figure 2:**
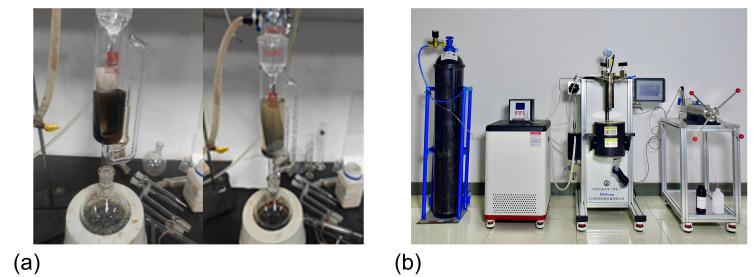
Experimental system for sedimentary sand treatment: (**a**) Soxhlet extraction system; and (**b**) supercritical water oxidation system.

**Figure 3:**
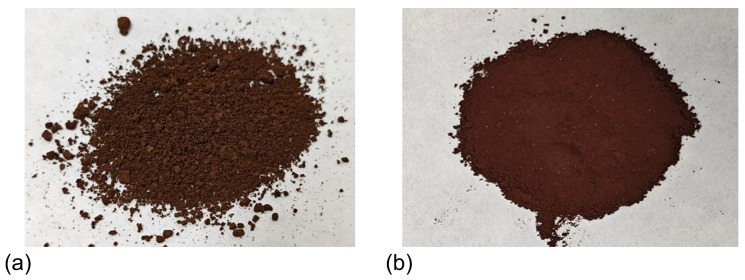
Characteristics of the experimental sand samples: (**a**) Soxhlet extraction sand samples; and (**b**) supercritical water oxidation sand samples.

To investigate the impact of polymers on sand sedimentation characteristics, Soxhlet extraction and supercritical water oxidation technologies were used to treat the sedimented sand. By combining a laser particle size analysis system, the polymer-containing sand particle size distribution and true sand particle size distribution in shale oil production fluids were analyzed. This helped reveal the mechanism of sand aggregation in regard to these fluids. The Soxhlet extraction experimental system, as shown in Figure 2a, consists of a condenser, an extraction tube, a siphon tube, and an extraction flask. Petroleum ether is used for cyclic extraction to remove the oil phase and some polymers, preserving the original aggregation morphology of the sand particles. The supercritical water oxidation experimental system, shown in Figure 2b, consists of a reactor, a condenser, a manual metering pump, and gas cylinders. Under excess oxidant and time conditions, the polymers are thoroughly degraded at 400 °C and 25 MPa to obtain the residue-free true sand particle size. The morphological characteristics of the sand samples treated using both methods are shown in Figure 3. Particle size testing was conducted using a Malvern Mastersizer 3000 laser particle size analyzer (Malvern Panalytical, Shanghai, China), based on the Mie scattering principle (detection range: 0.01–3500 μm). Wet ultrasonic dispersion was employed to ensure the accuracy of the data.

**Figure 4:**
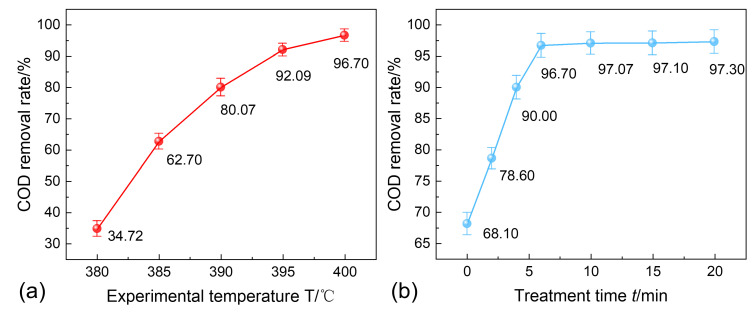
COD removal rate for different SCWO treatment conditions: (**a**) 25 MPa, 6 min; and (**b**) 25 MPa, 400 °C.

The chemical oxygen demand (COD) removal rates of the SCWO for different experimental temperatures and treatment times, subject to a pressure of 25 MPa, are shown in Figure 4. As indicated by Figure 4a,b, under conditions of 400 °C, 25 MPa, and 6 min, the COD removal rate can reach approximately 97%, and the COD removal rate curve stabilizes after 6 min, suggesting that the reaction has reached equilibrium at this stage. Therefore, it can be concluded that under these conditions (400 °C, 25 MPa, and 6 min), HPAM and other organic substances achieve satisfactory removal performance.

### 2.2. Materials

**Figure 5:**
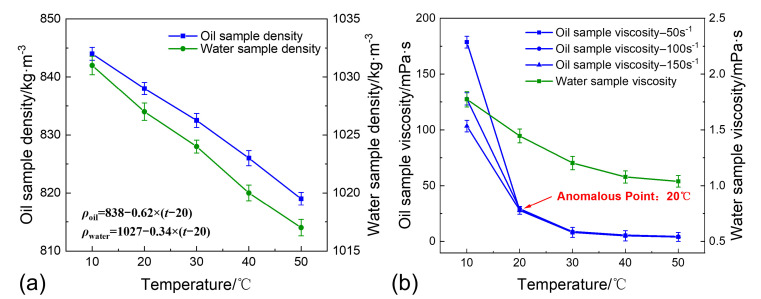
Physical properties of shale oil and produced water: (**a**) the density–temperature relationship; and (**b**) the viscosity–temperature relationship.

The experimental materials selected are shale oil and produced water from typical blocks at Changqing Oilfield (Xi’an, China). The produced water contains the polymer HPAM, with a molecular weight of 8 million and a hydrolysis degree from 25% to 30%. The polymer concentration in the produced water was determined to be 1250 mg∙L^−1^, using a chemiluminescence nitrogen determination method. The density–temperature and viscosity–temperature relationships of the shale oil and produced water samples are shown in Figure 5. As the temperature increases, both the density of the shale oil and the produced water decrease linearly, with a small change in density between 10 °C and 50 °C, as shown in Figure 5a. As the temperature increases, the stability of the polymers in the produced water decreases, and some polymers precipitate, leading to a reduction in the viscosity of the produced water, as shown in Figure 5b. Between 10 °C and 30 °C, as the temperature increases, the viscosity of the shale oil decreases rapidly, with an anomaly point near 20 °C. After 30 °C, the viscosity change tends to stabilize, as shown in Figure 5b.

**Figure 6:**
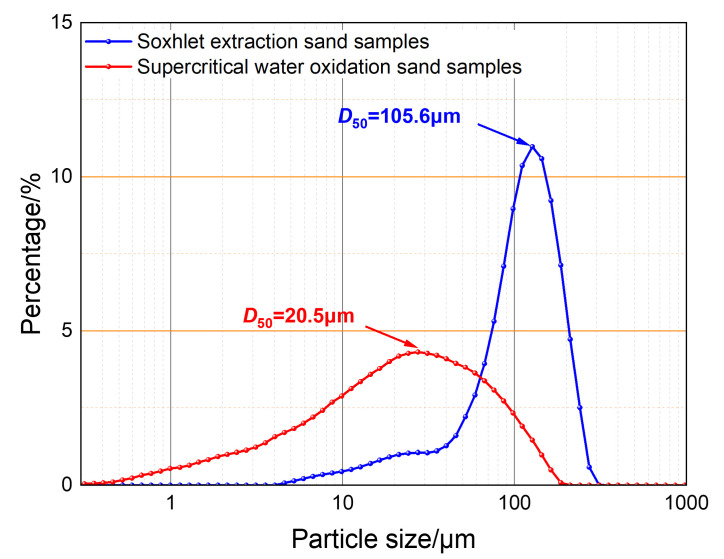
Particle size distribution of prepared sand sample.

Quartz sand, with a density of 2500 kg/m^3^, was selected as the experimental sand, based on the sand production properties of the shale oil reservoir. Fully sedimented sand samples from shale oil and the produced water from typical blocks at Changqing Oilfield were taken and successively treated using Soxhlet extraction and supercritical water oxidation technologies to restore the sedimented sand samples to their original dispersed state in the reservoir. The true particle size distribution of the sand produced from the reservoir was obtained using a Malvern Mastersizer 3000 laser particle size analyzer, as shown in Figure 6. According to the true particle size distribution of the sand produced from the reservoir, quartz sand was carefully sieved into different particle size ranges using standard sieves, and the sand samples for the sedimentation experiments were prepared by evenly mixing them in equal proportions.

### 2.3. Experimental Procedure

**Figure 7:**
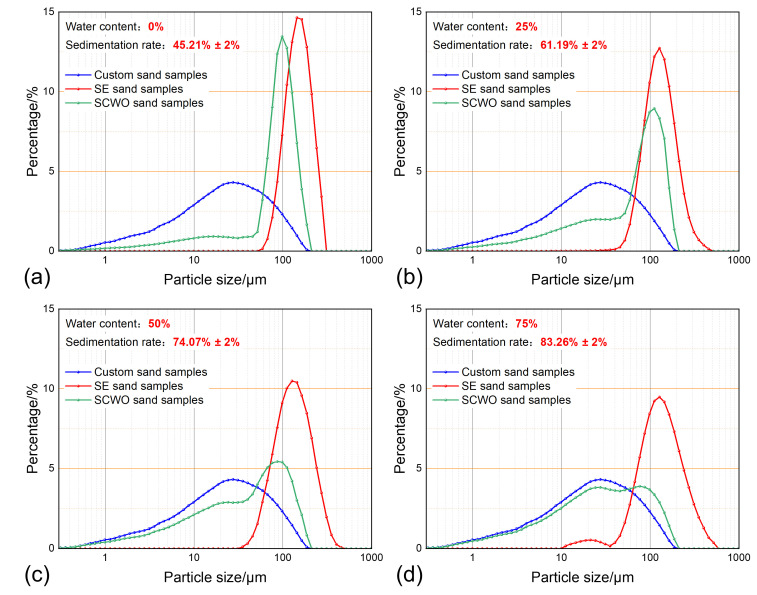
Sand sedimentation characteristics for different water content: (**a**) water content 0%; (**b**) water content 25%; (**c**) water content 50%; and (**d**) water content 75%.

**Figure 8:**
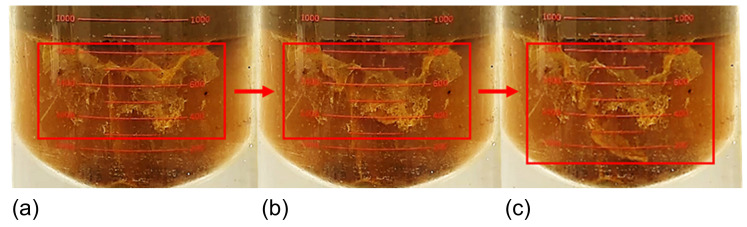
Flocculation–sedimentation effect: (**a**) 20 min; (**b**) 25 min; and (**c**) 30 min.

**Figure 9:**
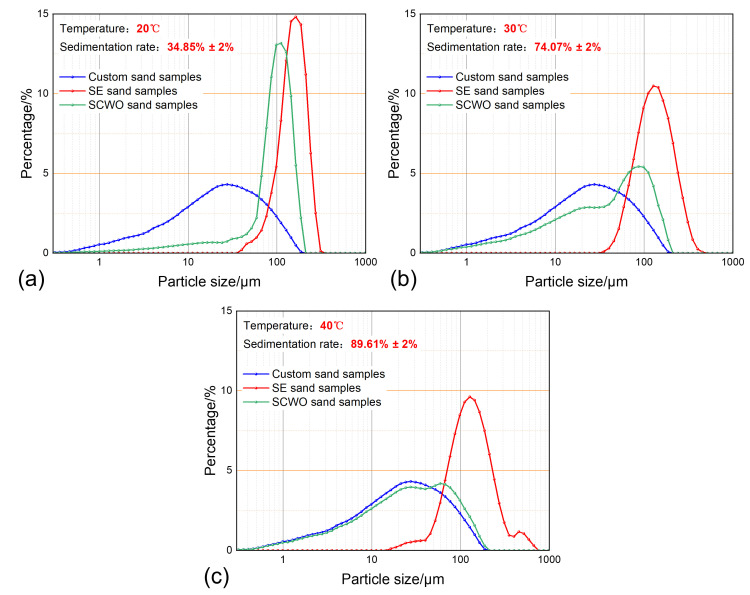
Sand sedimentation characteristics at different temperatures: (**a**) temperature 20 °C; (**b**) temperature 30 °C; and (**c**) temperature 40 °C.

**Figure 10:**
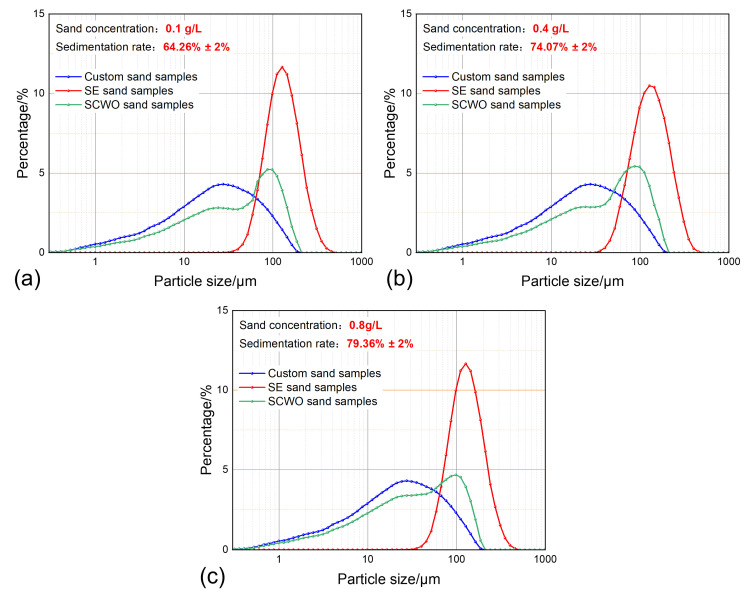
Sand sedimentation characteristics for different sand concentrations: (**a**) sand concentration 0.1 g/L; (**b**) sand concentration 0.4 g/L; and (**c**) sand concentration 0.8 g/L.

**Figure 11:**
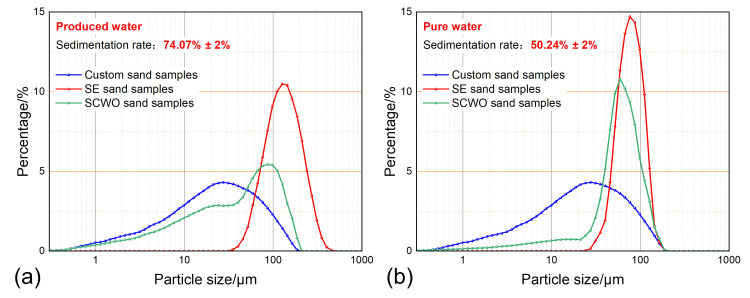
Sand sedimentation characteristics for different water phase properties: (**a**) produced water; and (**b**) pure water.

To investigate the sand sedimentation characteristics of polymer-containing shale oil production fluids, this study used the true operating conditions at the Changqing Oilfield. The experimental conditions are summarized in Table 1. The sand sedimentation curves corresponding to these conditions, shown in Figure 7, Figure 8, Figure 9, Figure 10 and Figure 11, represent the average results from multiple experiments. Each curve has a 5% error margin. Emulsion preparation was conducted using an IKA RW20 electric stirrer, IKA, Staufen, Germany (1000 rpm, 20 min) to simulate the on-site flow shear conditions, ensuring that the oil–water emulsification level matched the true state of the produced fluid. The settling time was set to 30 min, matching the operation time of the on-site separation process.

Before the experiment, a thermostatic water bath circulation system was used to synchronize the water bath tank and the experimental media (shale oil, produced water) in regard to the target temperature. Then, shale oil, produced water, and the sieved sand samples were mixed in the required proportions, and an IKA RW20 stirrer was used to prepare a uniform emulsion. The prepared emulsion was transferred to the sedimentation tank and allowed to stand for 30 min under constant temperature conditions to simulate the sedimentation conditions in the separator.

Soxhlet extraction and supercritical water oxidation methods were successively used to treat the sedimented sand. In regard to the Soxhlet extraction method, approximately 250 g of petroleum ether and the sedimented sand sample were placed into a 500 mL flask, with the flask connected to a Soxhlet extractor. The petroleum ether was used for cyclic extraction to remove the oil phase and some polymers. After the surface petroleum ether had evaporated, the sample was placed in an oven at 65 °C for 30 min to dry the sample, and was then weighed, thus obtaining the Soxhlet-extracted sand sample containing polymers. The treated sand sample was dried at 120 °C for 15 min, and its particle size distribution was measured using a Malvern Mastersizer 3000 laser particle size analyzer.

Next, the supercritical water oxidation method was used. The experimental sand sample and a certain volume of water (46–63 mL) were placed in the reactor, and the reaction system was sealed and its tightness was checked. A high-purity N_2_ purge system was used for 10 min to ensure the reaction occurred in an inert environment. The system then began to heat up, and when the reactor temperature reached the set value, a high-pressure metering pump injected a certain amount of 30 wt% H_2_O_2_ solution into the reactor, at which point the reaction timer started. After 6 min of reaction, the outlet valve was immediately opened, and the solid-phase products were separated and collected through the cooling system, obtaining the supercritical water oxidation-treated sand sample free of polymers. The treated sand sample was dried at 120 °C for 15 min, and its particle size distribution was measured using the Malvern Mastersizer 3000 laser particle size analyzer. The mass was weighed using an analytical balance to calculate the sedimentation rate. The sedimentation rate calculation formula is as follows:

(1) $$\eta =\frac{m}{M}\times 100\%$$

where *η* is the sedimentation rate, *M* is the mass of the sand sample before sedimentation (kg), and *m* is the mass of the sand sample after treatment using supercritical water oxidation (kg).

## 3. Results and Discussion

### 3.1. The Impact of the Water Content on the Sand Sedimentation Characteristics

At a temperature of 30 °C, a sand concentration of 0.4 g/L, and sedimentation time of 30 min, the sand sedimentation characteristics for different water content are shown in Figure 7. The experiment shows that as the water content increases, the sand sedimentation rate significantly improves, with the increase in the proportion of small particle-sized sand sedimentation being particularly noticeable. At the same time, as the water content increases, the proportion of agglomerates with a particle size greater than 300 μm in the Soxhlet-extracted sand gradually increases, exceeding the particle size of the prepared sand sample. After the supercritical water oxidation treatment decomposes the polymers in the sedimented sand, large aggregates bonded by polymers break down into fine sand particles, with a size range of 1–50 μm. As the water content increases, the particle size distribution curve of the SCWO-treated sand increasingly aligns with the particle size distribution curve of the prepared sand.

Analyzing the evolution of sand sedimentation characteristics, at 0% water content, the sedimentation mechanism mainly involves the single-particle sedimentation of large sand particles. This results in a concentrated particle size distribution, as shown in Figure 7a. When the water content increases to 25%, the sand sedimentation rate increases by about 16%. This is because as the water content increases, more sand particles enter the low-viscosity water phase during the emulsion preparation shear process, significantly reducing the viscous resistance to sedimentation, especially for smaller sand particles with poor sedimentation ability. In Figure 7b, this is reflected by the particle size distribution of the Soxhlet-extracted sand moving towards smaller particle sizes. At the same time, polymer precipitation occurs during the static sedimentation process, as shown in Figure 8. Flocculent polymers adhering to sand particles induce a flocculation–sedimentation effect, promoting the aggregation of small-sized sand particles into larger aggregates and settling synergistically, leading to a significant increase in the proportion of small particle-sized sand in the SCWO-treated sand. When the water content increases to 50% and 75%, the water phase in regard to the emulsion gradually becomes the continuous phase, and the viscous effect of the polymer starts to appear, slowing down the sedimentation rate of the sand. As a result, the sedimentation rate increases by 12.88% and 9.19%, with the increase showing a gradual deceleration trend. Additionally, the large amount of polymers dissolved in the sample with a high water content further enhances the flocculation–sedimentation effect, causing the sand particles to aggregate into clusters, with a particle size greater than 500 μm.

Water content affects sand sedimentation characteristics by adjusting the rheology and polymer content of the production fluid. As the water content increases, on the one hand, it enhances the water-washing effect of the produced water on the oil phase, promoting sand particles to enter the water phase and accelerating sedimentation. However, when the water content is too high, the hindering effect of the polymer can weaken the effectiveness of the water-washing effect. On the other hand, a high water content increases the polymer content in the production fluid, significantly increasing the likelihood of polymer precipitation adhering to sand particles, promoting the formation of large aggregates and enhancing the flocculation–sedimentation effect. As seen in Figure 7, the minimum particle size in the Soxhlet extraction-treated sand curve represents the smallest particle size that can be sediment under the current water-washing conditions. As the water content changes, the increase in the proportion of small particle-sized sand in the Soxhlet extraction-treated sand is much smaller than that in the SCWO-treated sand. This indicates that the polymer flocculation–sedimentation effect plays a greater role in assisting the sedimentation of small particle-sized sand.

### 3.2. The Impact of Temperature on Sand Sedimentation Characteristics

With a water content of 50%, a sand concentration of 0.4 g/L, and a sedimentation time of 30 min, the sand sedimentation characteristics at different temperatures are shown in Figure 9. The experimental results show that as the temperature increases, the sand sedimentation rate significantly improves. The particle size distribution curve of the SCWO-treated sand increasingly aligns with the particle size distribution curve of the prepared sand, while the particle size distribution range of the Soxhlet extraction-treated sand continues to expand and exhibits a bimodal feature in high-temperature conditions.

As seen in Figure 5b, at 20 °C, the viscosity of shale oil is high, resulting in greater viscous resistance to sand sedimentation. Additionally, the polymer stability is higher in low-temperature environments, and no flocculation–sedimentation occurs during static sedimentation. The particle size distribution curves of the Soxhlet extraction-treated sand and SCWO-treated sand both show a concentrated unimodal form, indicating that the sedimentation mechanism mainly involves single-particle sedimentation of large particle-sized sand, as shown in Figure 9a. When the temperature increases to 30 °C, the sand sedimentation rate increases by 39.22%. This is because the temperature rise causes the viscosity of shale oil to decrease, reducing viscous resistance and allowing more small particle-sized sand to sediment. The particle size distribution of the Soxhlet extraction-treated sand broadens towards smaller particle sizes. Meanwhile, the increase in temperature reduces the stability of the polymer in the produced fluid, leading to polymer precipitation and triggering the flocculation–sedimentation effect. As the sand particles aggregate, the particle size distribution of the Soxhlet extraction-treated sand broadens towards larger particle sizes. At the same time, the proportion of small particle-sized sand in the SCWO-treated sand increases significantly, indicating that the polymer flocculation–sedimentation effect plays a greater role in assisting small particle-sized sand sedimentation, as shown in Figure 9b. When the temperature further increases to 40 °C, the sand sedimentation rate increases by 15.54%. The decrease in shale oil viscosity allows more small particle-sized sand to sediment, and the proportion of small particle-sized sand in the Soxhlet extraction-treated sand increases further. The stability of the polymers in the produced fluid decreases further, leading to more precipitation of flocculent materials. The flocculation–sedimentation effect even produces aggregates with particle sizes greater than 600 μm, and the particle size distribution curve of the Soxhlet extraction-treated sand exhibits a bimodal phenomenon. Additionally, the flocculation–sedimentation effect helps more small particle-sized sand to aggregate and sediment, and the particle size distribution curve of the SCWO-treated sand increasingly approaches the particle size distribution curve of the prepared sand.

Temperature affects sand sedimentation characteristics by altering the rheology and polymer stability of the produced fluid. On the one hand, as the temperature increases, the viscosity of the produced fluid decreases, reducing the viscous resistance and promoting sand sedimentation. On the other hand, increasing the temperature disrupts polymer stability, promoting polymer precipitation and the formation of large particle-sized aggregates, which enhances the flocculation–sedimentation effect. Furthermore, the flocculation–sedimentation effect plays a greater role in assisting the sedimentation of small particle-sized sand.

### 3.3. The Impact of the Sand Concentration on Sand Sedimentation Characteristics

At a temperature of 30 °C temperature, a water content of 50%, and a sedimentation time of 30 min, the sand sedimentation characteristics at different sand concentrations are shown in Figure 10. The experiment shows that as the sand concentration increases, the sand sedimentation rate slightly improves. Additionally, the particle size distribution curves of the Soxhlet extraction-treated sand and SCWO-treated sand do not show a significant correlation with changes in the sand concentration. Under 50% water content conditions, when the sand concentration increases by eight times, the sand sedimentation rate increases by only 15.09%, and there is no significant change in the sedimented sand particle size distribution. This indicates that the sand concentration in the emulsion has only a secondary impact on the sand sedimentation characteristics.

### 3.4. Water-Washing Effect Evaluation

The water-washing effect during the sand sedimentation process is evaluated by using pure water to remove the influence of polymers on the produced water. Emulsions were prepared using pure water and produced water for comparative experiments. At a temperature of 30 °C temperature, a water content of 50%, a sand concentration of 0.4 g/L, and a sedimentation time of 30 min, the sand sedimentation characteristics with different water phase properties are shown in Figure 11. The experiment shows that under the same sedimentation conditions, the sand sedimentation rate of the emulsion with produced water containing polymers increases by 23.83% compared to the pure water emulsion.

Comparing Figure 11a,b, it can be seen that under pure water conditions, the particle size distribution curves of the Soxhlet extraction-treated sand and SCWO-treated sand both show a concentrated unimodal form, indicating that the sedimentation mechanism mainly involves single-particle sedimentation of large particle-sized sand. This is because under pure water conditions, the polymer concentration in the emulsion is very low, and there is no flocculation effect to increase the sand particle size. Therefore, no sand particles larger than 300 μm are present in the Soxhlet extraction-treated sand particle size distribution curve. Moreover, under pure water conditions, the minimum particle size of the Soxhlet extraction-treated sand is slightly smaller than that in produced water, indicating that the water-washing effect in pure water conditions allows smaller particle-sized sand to sediment. This is because the lack of polymer hindrance in the pure water environment results in a more effective water-washing effect. However, in regard to the particle size distribution curve of the SCWO-treated sand, the proportion of small particle-sized sand in pure water conditions is much smaller than that in produced water. This indicates that the polymer flocculation–sedimentation effect plays a more significant role in promoting the sedimentation of small particle-sized sand, and it is this effect that leads to a further improvement in the sedimentation rate.

### 3.5. Analysis of Sand Sedimentation Mechanisms

(1) Polymer flocculation–sedimentation mechanism

Based on the particle size distribution characteristics of Soxhlet extraction-treated and SCWO-treated sand, the median particle size *D* of the particle distribution is used to represent the overall particle size distribution of the sedimented sand [48]. To quantitatively characterize the enhanced effect of polymer flocculation–sedimentation on sand particles, the aggregation coefficient *K* is defined as:

(2) $$K=\frac{D_{M}-D_{S}}{D_{S}}\times 100\%$$

where *D_M_* is the median particle size of the Soxhlet extraction-treated sand, μm; and *D_S_* is the median particle size of the SCWO-treated sand, μm.

**Figure 12:**
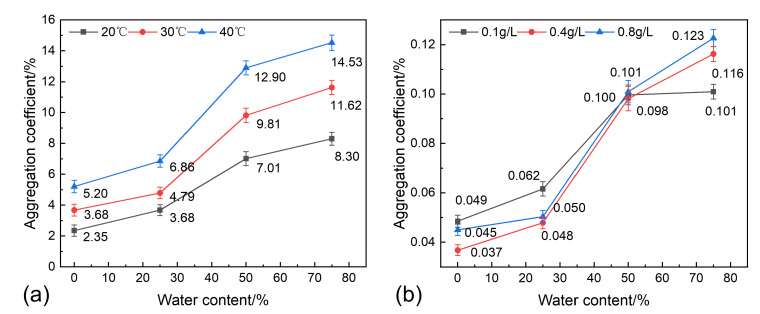
Aggregation coefficients for different conditions: (**a**) aggregation coefficient at different temperatures; (**b**) aggregation coefficient at different sand concentrations.

By using the aggregation coefficient to represent the particle size differences between the two treatment methods, the influence of the water-washing effect can be effectively removed, and the polymer flocculation–sedimentation effect can be quantified. The characteristics of the aggregation coefficient at different temperatures, sand concentrations, and water content conditions are shown in Figure 12.

The variation in the aggregation coefficient according to the water content under different temperatures is shown in Figure 12a. For 0% water content (pure oil condition), the polymer content is low, and the aggregation coefficient increases linearly with the temperature. At 40 °C, the aggregation coefficient is only 5.2%, and the sedimentation mechanism is primarily gravity-driven single-particle sedimentation. When the water content increases to 25%, the dissolved polymers in the produced water increase the overall polymer content in the emulsion, but the polymer flocculation–sedimentation effect is still not significant. At 40 °C, the aggregation coefficient is 6.8%. For the 50% water content condition, the polymer flocculation–sedimentation effect is significantly enhanced, and the precipitation of the aggregates increases noticeably. At 40 °C, the aggregation coefficient is 12.9%, an increase of 6.1% compared to the 25% water content condition. When the water content increases to 75%, the polymer content tends to saturate, and the increase in the aggregation coefficient slows down. The variation in the aggregation coefficient according to the water content under different sand concentration conditions is shown in Figure 12b. For the same water content, the aggregation coefficient tends to be consistent at different sand concentrations, indicating that the sand concentration has a minor effect on the precipitation of the aggregates. Therefore, the flocculation–sedimentation effect is primarily influenced by the water content and temperature. Within the 20–40 °C range, the flocculation–sedimentation effect increases linearly with the temperature, and within the 25–50% water content range, the increase in the flocculation–sedimentation effect is particularly significant.

To more effectively assess the proposed polymer flocculation–sedimentation mechanism, the Stokes sedimentation model [13] is introduced to analyze the influence of polymer flocculation on the sedimentation rate. The model is expressed as follows:

(3) $$V=\frac{gD^{2}\left(\rho_{P}-\rho_{L}\right)}{18\mu_{L}}$$

where *V* is the sedimentation velocity, m/s; *g* is the gravitational acceleration, m/s^2^; *D* is the particle diameter, m; *ρ*_P_ is the particle density, kg/m^3^; *ρ*_L_ is the liquid phase density, kg/m^3^; and *μ*_L_ is the liquid phase viscosity, Pa·s.

**Figure 13:**
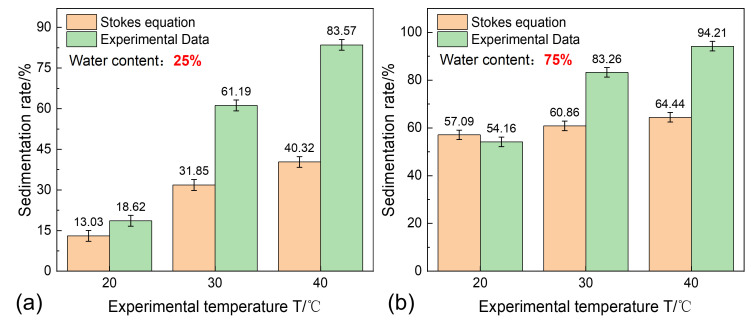
The characteristics of flocculation–sedimentation: (**a**) water content 25%; and (**b**) water content 75%.

For the conditions with a water content of 25% and 75%, assuming that the emulsion containing sand is uniformly distributed, the critical particle diameters that can settle 30 cm within 30 min, are calculated using the Stokes model, based on the properties of oil and water. The ideal sedimentation rates are then calculated according to the original sand particle size distribution, and the model results are compared with the experimental results, as shown in Figure 13. The analysis shows that at 20 °C, there is no significant flocculation–sedimentation phenomenon, and the deviation between the Stokes model-calculated sedimentation rate and the experimental value is within ±5%. As the temperature increases, the density and viscosity of the oil–water phase decrease, reducing the viscous resistance against sedimentation and increasing the settling velocity. The Stokes model calculations for the sedimentation rates increase under all the water content conditions. At the same time, higher temperatures reduce the stability of the polymer, promoting flocculation–sedimentation, which results in a substantial increase in the experimental sedimentation rates for all the water contents. Under the 30 °C condition, the average experimental data exceeds the Stokes model calculation by 25.87%, while at 40 °C, it exceeds the calculation by 36.51%. The flocculation–sedimentation effect shows a linear growth trend with rising temperatures. Furthermore, at 40 °C and 25% water content, flocculation–sedimentation leads to a 43.25% increase in the sedimentation rate, while under the 75% water content condition, the increase is only 29.77%. This also demonstrates that the flocculation–sedimentation phenomenon becomes saturated as the water content increases.

(2) Synergistic sedimentation mechanism of the temperature and water content

**Figure 14:**
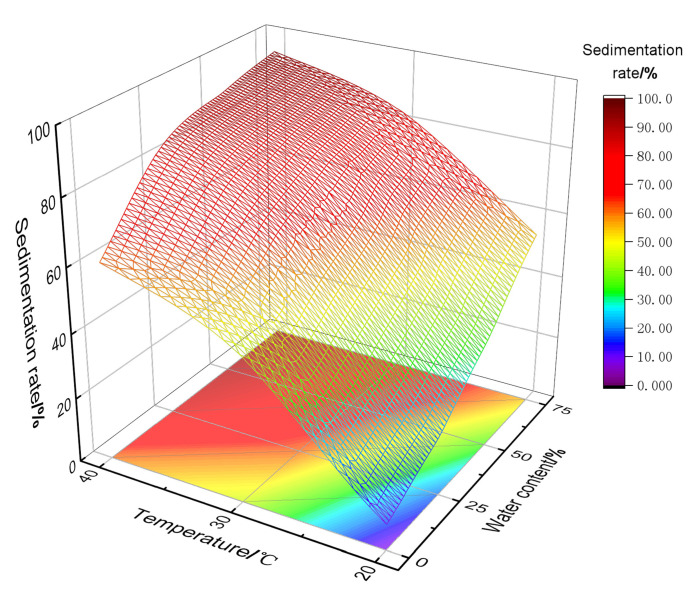
Correlation laws among sand sedimentation rate, temperature, and water content.

The analysis of the experimental results shows that the temperature and water content regulate sand sedimentation behavior by altering the rheology, polymer stability, and content of polymer-containing shale oil production fluids. As shown in Figure 14, the correlation between the sand sedimentation rate, temperature, and water content is analyzed using the response surface methodology. It is found that the sand sedimentation rate is positively correlated with both the temperature and water content, with temperature being the dominant factor. The water content indirectly influences the sand sedimentation characteristics by regulating the water-washing effect and flocculation–sedimentation effect. Under low-temperature conditions, the high viscosity of shale oil results in greater viscous resistance to sand particle sedimentation. The sedimentation mechanism is dominated by single-particle gravity sedimentation, and even in high water content conditions, the sand sedimentation rate is only 50%. Increasing the temperature significantly reduces the viscosity of shale oil, promoting sand particle sedimentation. However, when the temperature exceeds 30 °C, the reduction in shale oil viscosity slows down, which causes a sharp decline in polymer stability, accelerating polymer precipitation. This strengthens the flocculation–sedimentation effect, gradually shifting the sedimentation mechanism towards multi-particle flocculation–sedimentation.

Therefore, the sand particle sedimentation characteristics of polymer-containing shale oil production fluids are influenced by two synergistic mechanisms: one is gravity-dominated single-particle gravity sedimentation, and the other is multi-particle flocculation–sedimentation, induced by polymer precipitation and particle adhesion. Based on this, by adjusting parameters such as the temperature, water content, and sand concentration in the gathering system, and suppressing polymer precipitation to improve the suspension stability of sand particles in the production fluid, the risk of sedimentation-induced transportation issues in pipelines can be effectively reduced.

## 4. Conclusions

This study used Soxhlet extraction and supercritical water oxidation technologies to obtain Soxhlet extraction-treated sand samples containing polymers and supercritical water oxidation-treated sand samples without polymers, and compared the sedimentation characteristics of the sand in the production fluids under the influence of polymers. The experiments demonstrated that the sand sedimentation process in polymer-containing shale oil production fluids involves two mechanisms: gravity-dominated single-particle sedimentation and polymer-induced multi-particle flocculation–sedimentation. Both of these mechanisms are influenced by the characteristics of the production fluid. The conclusions are as follows:

(1) Water content is a key factor in regulating sand sedimentation characteristics. As the water content increases, sand particles are more easily transferred into the low-viscosity water phase during sedimentation, significantly reducing the viscous resistance to sedimentation. However, when the water content is too high, the polymers in the continuous water phase hinder sand sedimentation, weakening the water-washing effect. On the other hand, a high water content increases the polymer content in the produced fluid, significantly increasing the likelihood of polymer precipitation adhering to sand particles, enhancing the flocculation–sedimentation effect;
(2) Temperature is the core governing factor in regulating sand sedimentation characteristics. Increasing the temperature significantly reduces the viscosity of shale oil, weakening the viscous resistance to sand particle sedimentation and enhancing single-particle gravity sedimentation. On the other hand, increasing the temperature disrupts polymer stability, promoting polymer precipitation, enhancing the flocculation–sedimentation effect, and strengthening multi-particle flocculation–sedimentation;
(3) The sand concentration is a secondary influencing factor on sand sedimentation characteristics. The experiment shows that under 50% water content conditions, when the sand concentration increases by eight times, the sand sedimentation rate increases by only 15.09%, and the particle size distribution curves of Soxhlet extraction-treated sand and SCWO-treated sand show no significant correlation with the changes in the sand concentration. Therefore, it can be concluded that, with the sand sample particle size distribution remaining unchanged, the sand particle management and regulation in polymer-containing shale oil production fluids can to some extent ignore the risk of fluctuations in sand concentration;
(4) The water-washing effect under pure water conditions shows that, under the same sedimentation conditions, the proportion of small particle-sized sand under pure water conditions is much smaller than that under produced water conditions, indicating that the polymer flocculation–sedimentation effect plays a more significant role in promoting the sedimentation of small particle-sized sand. Therefore, it can be concluded that achieving the sedimentation of small particle-sized sand is key to sand particle management and regulation of polymer-containing shale oil production fluids;
(5) In summary, in regard to the sand removal conditions of a three-phase separator involving shale oil with polymer-containing produced liquid, it is advisable to appropriately increase the water-washing stage and increase the separation temperature to promote sand particle sedimentation. In regard to pipeline transportation conditions for shale oil with polymer-containing produced liquid, the water content should be minimized, and the transportation temperature should be reduced to maintain the suspension stability of the sand particles.

## Figures and Tables

**Table 1:** Experiment design conditions table.

Group Number	Water Content	Temperature	Sand Concentration	Sedimentation Time	Water Phase Property	Related Figure
1	0%	30 °C	0.4 g/L	30 min	produced water	Figure 7a
2	25%	30 °C	0.4 g/L	30 min	produced water	Figure 7b
3	50%	30 °C	0.4 g/L	30 min	produced water	Figure 7c
4	75%	30 °C	0.4 g/L	30 min	produced water	Figure 7d
5	50%	20 °C	0.4 g/L	30 min	produced water	Figure 9a
6	50%	40 °C	0.4 g/L	30 min	produced water	Figure 9c
7	50%	30 °C	0.1 g/L	30 min	produced water	Figure 10a
8	50%	30 °C	0.8 g/L	30 min	produced water	Figure 10c
9	50%	30 °C	0.4 g/L	30 min	pure water	Figure 11b

## Data Availability

The original contributions presented in this study are included in the article. Further inquiries can be directed to the corresponding authors.
